# First detection of clade 2.3.4.4b H5N1 highly pathogenic avian influenza virus in a wild leopard cat (*Prionailurus bengalensis*) in South Korea

**DOI:** 10.3389/fvets.2025.1638067

**Published:** 2025-07-29

**Authors:** Young-Jae Si, Sun-Hak Lee, Dong-Ju Kim, Kwanghee Lee, Min-a Lee, Dong-Yeop Lee, Ye-Ram Seo, Hyesung Jeong, Suwoong Lee, Dong-Hun Lee

**Affiliations:** ^1^Wildlife Disease Research Team, National Institute of Wildlife Disease Control and Prevention, Gwangju, Republic of Korea; ^2^Wildlife Health Laboratory, College of Veterinary Medicine, Konkuk University, Seoul, Republic of Korea; ^3^Avian Disease Laboratory, College of Veterinary Medicine, Konkuk University, Seoul, Republic of Korea

**Keywords:** HPAI H5N1, wild leopard cat, South Korea, clade 2.3.4.4b, mammalian adaptation, zoonotic potential

## 1 Introduction

The A/goose/Guangdong/1/1996 (Gs/GD) lineage of highly pathogenic avian influenza (HPAI) viruses initially identified in China in 1996 and have evolved over subsequent decades, characterized by accumulation of point mutations and multiple reassortment events with low pathogenic avian influenza (LPAI) viruses (1, 2). Particularly those of the H5N1 subtype within clade 2.3.4.4b have emerged as a significant threat to poultry, wild birds, and mammals worldwide (3). Since their widespread dissemination in wild bird population in the early 2020s, these viruses have caused multiple sporadic infections in mammals, including small carnivores, marine mammals, cattle, and humans (3–6). These cross-species transmissions, often linked to the consumption of infected birds or exposure to contaminated environments, have raised significant concerns about the zoonotic potential of clade 2.3.4.4b H5N1 and its capacity to evolve into a strain with pandemic potential.

Since its initial detection in 2014 with the H5N8 subtype in wild birds, multiple subtypes of clade 2.3.4.4b highly pathogenic avian influenza viruses (HPAIVs) have caused recurrent outbreaks in South Korea including H5N1, H5N6, and H5N8 (7). Notable epidemics occurred during 2021–2022, with 44 cases primarily of H5N1 identified in wild birds (7), and 2022–2023, with 174 wild bird cases, highlighting its persistent circulation wild waterfowl population (8). These outbreaks underscore the role of wild birds in introduction and spread of the virus across South Korea, posing ongoing challenges to poultry industry and public health. In addition, although HPAIV infection in domestic cats were reported in 2016 and 2023 (9–11), no instances of wild mammalian infection were documented in South Korea during these epidemics.

Here, we report the first documented case of H5N1 HPAI in a wild mammal in South Korea, identified in a leopard cat (*Prionailurus bengalensis*). On March 18, 2025, a wild leopard cat discovered moribund near a freshwater reservoir in Hwasun County of Jeollanam-do Province and submitted to the National Institute of Wildlife Disease Control and Prevention (NIWDC) of Korea. We isolated the H5N1 virus from this leopard cat, sequenced, and assessed its evolutionary history and molecular markers indicative of mammalian adaptation.

## 2 Materials and methods

### 2.1 Sample collection and virus isolation

On March 18, 2025, a wild leopard cat was found moribund near the reservoir in Hwasun County, Jeollanam-do Province, South Korea (GPS coordinates ≈ 35°03′N, 126°59′E). The wild leopard cat transported to the Jeollanamdo wild animal rescue center and died within a few hours. The carcass was submitted to the biosafety level 3 facility of NIWDC and organs including brain, trachea, and lung were collected. Samples were placed in phosphate-buffered saline with 400 mg/ml gentamicin, homogenized by vortexing, and filtered using a 0.45-μm Minisart Syringe Filter (Sartorius, Göttingen, Germany) following centrifugation at 3,000 rpm for 10 min. Filtered supernatant was inoculated into 10-day-old specific-pathogen-free embryonated chicken eggs and incubated at 37°C for 72 h. Allantoic fluids were harvested and tested for hemagglutination activity (HA) using 0.5% chicken red blood cells. RNA was extracted from tissue samples and HA-positive allantoic fluids using the Maxwell RSC simply RNA Tissue Kit (Promega, Madison, WI, USA) and screened for influenza A matrix and H5 genes via real-time reverse transcription-PCR (rRT-PCR), following established protocols (12).

### 2.2 Whole genome sequencing and sequence analysis

Complementary DNA was synthesized using the SuperScript III First-Strand Synthesis System (Invitrogen, Carlsbad, CA, USA), and the eight gene segments were amplified with AccuPrime Pfx DNA Polymerase (Invitrogen, Carlsbad, CA, USA), as previously described (13). DNA libraries were prepared using the Illumina DNA Prep Kit (Illumina, San Diego, CA, USA) and sequenced on the Illumina MiSeq platform (paired-end 150 bp). Raw reads were trimmed using BBDuk (v38.84) with a minimum quality threshold of 30 (14), assembled *de novo* with SPAdes (v3.15.5). Trimmed reads were mapped to the top BLAST result from the GISAID EpiFlu database using Minimap2 (v2.24). Consensus sequences were generated using Geneious Prime software and deposited in GISAID Epiflu database (EPI_ISL_20051149).

### 2.3 Phylogenetic and mutation analysis

The top 250 hits for each segment query were retrieved and sequences with high identity (ranging from 99.5 to 99.9%, depending on the segment) were removed using CD-hit (15). We also included genome sequences of four H5N1 HPAIVs [A/Wild_Duck/Korea/24WF364-8P/2024, A/Eurasian_wigeon/Korea/24WF382-7P/2024, A/Vulture/Korea/24WC103/2024, and A/Bean_goose/Korea/24WC196/2025] which were isolated from wild birds during the winter season of 2024-2025, all of which have been deposited in GISAID with their respective accession numbers (EPI_ISL_20051150, 19832581-19832583). Phylogenetic trees were constructed for each gene segment using RAxML v8.0 with the general time reversible model and 1,000 bootstrap replicates (16). Interactive Tree of Life (iTOL) was used to visualize the tree of each gene (17). A Bayesian relaxed-clock phylogeny of the hemagglutinin (HA) gene was reconstructed using BEAST version 1.10.4 (18), employing the Hasegawa-Kishino-Yano substitution model with an uncorrelated log-normal distribution and a Gaussian Markov Random Field (GMRF) Bayesian skyride coalescent prior (19). The Markov Chain Monte Carlo (MCMC) process was executed in parallel across three chains, each comprising 50 million iterations, with results combined after a 10% burn-in. All parameters achieved effective sample sizes (ESS) >200 and were assessed using TRACER v1.5 (http://tree.bio.ed.ac.uk/software/tracer/) (20). A maximum clade credibility (MCC) tree was generated using TreeAnnotator and visualized with FigTree v1.4.4 (http://tree.bio.ed.ac.uk/software/figtree/).

Molecular markers of mammalian adaptation, pathogenicity, and drug resistance were identified using the FluMut tool (21). In the FluMut analysis, in addition to the sequence isolated from the leopard cat, seven of clade 2.3.4.4b HPAI H5N1 viruses were additionally analyzed including viruses reported in infected mammals in the United States (4), viruses isolated from domestic cats in Korea in 2023 (9), and viruses isolated from wild birds during the 2024–2025 winter season (22).

## 3 Descriptive results

### 3.1 Isolation and genome sequencing of the virus

The brain and trachea from the submitted leopard cat tested positive for influenza A virus via chicken embryonated egg inoculation and rRT-PCR. The isolated virus, designated A/Leopard Cat/Korea/24WM130/2025(H5N1) (hereafter 24WM130) yielded 288,814 NGS reads, enabling assembly of complete coding genome sequences across all eight influenza virus segments.

### 3.2 Genome analysis

**Figure 1:**
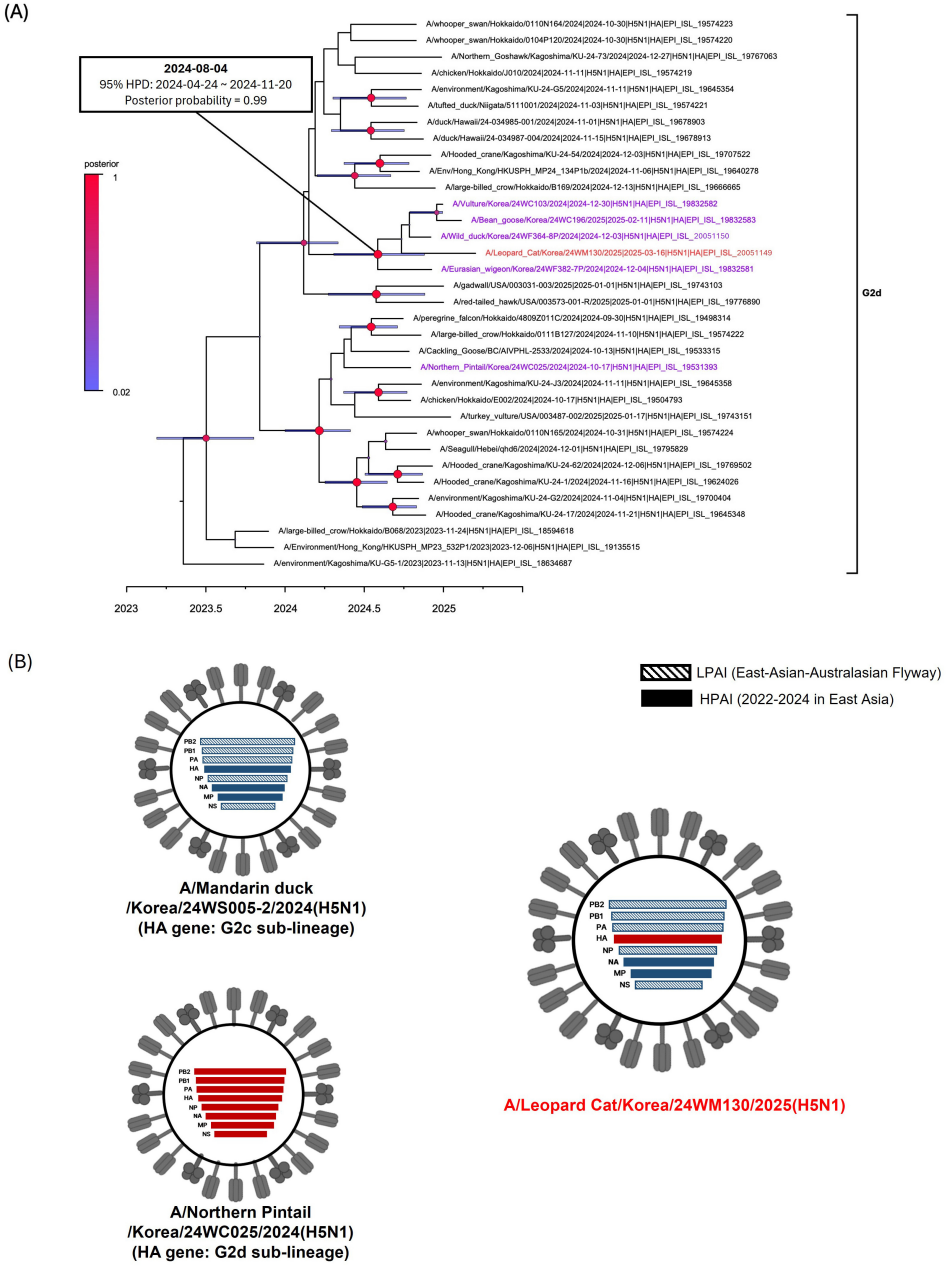
Phylogenetic analysis and genotypes of clade 2.3.4.4b H5N1 HPAI virus found in a wild leopard cat (*Prionailurus bengalensis*) in South Korea, March 2025. **(A)** Time-scaled Maximum clade credibility phylogeny constructed using the hemagglutinin gene sequence of G2d subgroup of H5N1 HPAI virus. Red taxa marked with circles represent the H5N1 isolate from the leopard cat in South Korea. Purple taxa indicate the H5N1 HPAI viruses isolated from wild birds during the 2024–2025 winter season. Node bars represent 95% HPD of the node height with a posterior probability >0.5. Node size and color correspond to posterior probability values. The horizontal axis represents the decimal year. The estimated time of the most recent common ancestry with 95% highest posterior density (HPD) is displayed on the node of cluster. **(B)** Schematic representation of the genotypes of H5N1 viruses from South Korea, 2024–2025. Bars represent eight gene segments of the avian influenza virus in the following order (top to bottom): polymerase basic 2, polymerase basic 1, polymerase acidic, hemagglutinin, nucleoprotein, neuraminidase, matrix, and non-structural. Different bar colors indicate different virus origins estimated from maximum-likelihood phylogenetic trees.

The virus was identified as HPAIV based on the presence of multiple basic amino acids at the HA proteolytic cleavage site (PLREKRRKR/G) (23). All gene segments of the 24WM130 virus clustered with clade 2.3.4.4b H5N1 HPAIVs isolated from wild birds in South Korea during the 2024–2025 winter season, showing close genetic relatedness and a likely origin from infected wild birds through predation or scavenging (Supplementary Figure 1). We previously reported two genotypes of H5N1 clade 2.3.4.4b viruses in October 2024, the genotype 1 and 2, represented by A/Northern pintail/Korea/24WC025/2024 virus and A/Mandarin duck/Korea/24WS005-2/2024 virus, respectively (22). Genotype 1 possessed a G2d-lineage HA gene and a genome constellation identical to strains circulating in Japan during 2023–2024. Genotype 2 carried a G2c-lineage HA gene, neuraminidase (NA) and M genes from H5Nx clade 2.3.4.4b viruses circulating in 2022–2024, and internal genes from LPAIVs in the East Asian–Australasian flyway. The 24WM130 virus had a G2d-lineage HA gene, while its remaining segments closely matched those of genotype 2, indicating a reassortant virus derived from early HPAI outbreaks in October 2024 (22) (Figure 1B). These reassortment events are likely driven by the high density and mobility of migratory birds, which promote co-infection and gene exchange. In the Bayesian phylogenetic analysis of the HA gene, the 24WM130 virus clustered with clade 2.3.4.4b H5N1 HPAIVs from wild birds in South Korea during December 2024–February 2025 and supported by a high posterior probability (0.99; Figure 1A). Their tMRCA was estimated to be August 2024 (95% BCI: April 24–November 20, 2024), suggesting that these viruses originated most likely during the late breeding season of waterfowl in Eurasia.

Leopard cat is an endangered, solitary, and opportunistic mesocarnivore species native to South Korea and other parts of Asia. Their solitary lifestyle, coupled with the absence of additional HPAI detections in other mammals, suggests that this outbreak was most likely a sporadic event. The leopard cat's ecological overlap with wild birds and free-ranging mammals raises concerns about potential disease spillovers and their role as an intermediate species in future pandemics (24–26).

Among the 36 mammalian adaptation markers identified in 24WM130, three mutations (Table 1), I292V in PB2, D154N in HA, and D74N in NS, were unique compared to other H5N1 viruses identified in mammals. The I292V in PB2 and D74N in NS substitutions have been associated with increased polymerase activity in mammalian hosts and enhanced virulence in mice. The D154N in HA has been reported to increase binding affinity to α2,6-linked sialic acid receptors (27, 28). Notably, the 24WM130 virus did not possess the E627K or D701N in PB2 which were previously identified in clade 2.3.4.4b HPAI H5N1 viruses isolated from domestic cats in South Korea and M631L in PB2 which was a unique mutation found in dairy cows in the U.S. The HPAI H5N1 viruses from wild birds in Korea during the same wintering season also had 35 mammalian adaptation markers out of 36 detected in the 24WM130, highlighting the high potential for clade 2.3.4.4b H5N1 viruses in wild birds to spillover to mammalian hosts. The D74N substitution, detected exclusively in the 24WM130 virus and absent from closely related avian viruses in Korea during 2024–2025, suggests it may have emerged in the leopard cat.

**Table 1:** Comparison of mammalian adaptation markers among A/Leopard Cat/Korea/WM130/2025, other mammalian-derived strains, and clade 2.3.4.4b highly pathogenic H5N1 viruses isolated from Korean wild birds in the same season.

**Gene**	**Mutation^a^**	**A/Leopard Cat/ Korea/24WM 130/2025**	**A/feline/South Korea/SNU-01/2023**	**A/feline/ Korea/ M305-3/ 2023**	**A/Mandarin duck/ Korea/24WS 005-2/2024**	**A/Northern pintail/Korea/ 24WC25/2024**	**A/Spot-billed duck/Korea/ 24WF364-8P**	**A/dairy cow/USA/ 007549-005/2025**	**A/Louisiana/ 12/2024**	**Effect^a^**
PB2	L89V	${\text{V}}^{\text{b}}$	**V**	**V**	**V**	**V**	**V**	**V**	**V**	• Increased polymerase activity in mammalian, virulence in mice
	I292V	V	I	I	**V**	I	**V**	I	I	
	G309D	D	**D**	**D**	**D**	**D**	**D**	**D**	**D**	
	T339K	K	**K**	**K**	**K**	**K**	**K**	**K**	**K**	
	K389R	R	**R**	**R**	**R**	**R**	**R**	**R**	**R**	
	R447G	G	**G**	**G**	**G**	**G**	**G**	**G**	**G**	
	K482R	K	K	K	K	**R**	K	K	K	
	I495V	V	**V**	**V**	**V**	**V**	**V**	I	**V**	
	V598T	T	**T**	**T**	**T**	**T**	**T**	**T**	**T**	
	M631L	M	M	M	M	M	M	**L**	M	
	A676T	T	**T**	**T**	**T**	**T**	**T**	A	**T**	
	E627K	E	E	**K**	E	E	E	E	E	• Increased polymerase activity, replication in mammalian • Increased virulence in mice, ferrets • Contributes to airborne pathogenicity in ferrets
	D701N	D	**N**	D	D	D	D	D	D	• Increased polymerase activity, replication in mammalian • Increased virulence in mice, guinea pigs
PB1	D3V	V	**V**	**V**	**V**	**V**	**V**	**V**	**V**	• Increased polymerase activity in mammalian
	F2:N66S	S	**S**	**S**	**S**	**S**	**S**	**N**	**S**	
	R207K	K	**K**	**K**	**K**	**K**	**K**	**K**	**K**	
	D622G	G	**G**	**G**	**G**	**G**	**G**	**G**	**G**	
PA	S37A	A	**A**	**A**	**A**	**A**	**A**	**A**	**A**	• Increased polymerase activity in mammalian
	N383D	D	**D**	**D**	**D**	**D**	**D**	**D**	**D**	
	N409S	S	**S**	**S**	**S**	**S**	**S**	**S**	**S**	
	K497R	K	**K**	**K**	**K**	**K**	**K**	R	**K**	
HA	S133A	A	**A**	**A**	**A**	**A**	**A**	**A**	**A**	• Increase virus binding to α2–6
	D154N	N	D	D	D	**N**	**N**	**N**	**N**	
	K218Q	Q	**Q**	**Q**	**Q**	**Q**	**Q**	**Q**	**Q**	
	S223R	R	**R**	**R**	**R**	**R**	**R**	**R**	**R**	
	S107R	R	**R**	**R**	**R**	**R**	**R**	**R**	**R**	• Increase virulence in mice
	T108I	I	**I**	**I**	**I**	**I**	**I**	**I**	**I**	
	T156A	A	**A**	**A**	**A**	**A**	**A**	**A**	**A**	• Increase virus binding to α2–6 • Increase transmission in guinea pigs
	N189D	N	N	N	**D**	N	N	N	N	• Increase virus binding to α2–6 • Transmissible among ferrets
NP	N319K	N	N	N	N	**K**	N	N	N	• Increased polymerase activity, replication in mammalian
MP (M1)	N30D	D	**D**	**D**	**D**	**D**	**D**	**D**	**D**	• Increased virulence in mice
	I43M	M	**M**	**M**	**M**	**M**	**M**	**M**	**M**	
	T215A	A	**A**	**A**	**A**	**A**	**A**	**A**	**A**	
NS (NS-1)	P3S	S	**S**	**S**	**S**	**S**	**S**	**S**	**S**	• Increase replication in mammalian
	R41K	K	**K**	**K**	**K**	**K**	**K**	**K**	**K**	
	K55E	E	**E**	**E**	**E**	**E**	**E**	**E**	**E**	
	K66E	E	**E**	**E**	**E**	**E**	**E**	**E**	**E**	
	C138F	F	**F**	**F**	**F**	**F**	**F**	**F**	**F**	
	P42S	S	**S**	**S**	**S**	**S**	**S**	**S**	**S**	• Increase virulence in mice
	D74N	N	D	D	D	D	D	D	D	• Increase virulence in mice
	I106M	M	**M**	**M**	**M**	**M**	**M**	**M**	**M**	• Increase replication in mammalian
	T48A	A	**A**	**A**	**A**	**A**	**A**	**A**	**A**	• Increase virulence in ferret

a Mammalian adaptation mutations and their effects were identified using the FluMut tool.

b Identified substitutions associated with mammalian adaptation are indicated in bold. Substitutions detected in A/Leopard Cat/Korea/WM130/2025 are highlighted in red.

## 4 Conclusion

The identification of clade 2.3.4.4b H5N1 in a wild leopard cat in South Korea highlights the evolving epidemiology of HPAI and the need for expanded surveillance in wild mammals. Although infections in domestic cats were reported in South Korea in 2016 and 2023 (9–11), this case represents the first confirmed detection of HPAI H5N1 in a wild mammalian species in the country. Given the role of wild birds in the spread and maintenance of HPAIVs, the significant presence of mammalian adaptation markers in viruses circulating in wild bird populations is of great concern, particularly for potential widespread dissemination and interspecies transmissions. Genomic data from this study, shared via GISAID, will support ongoing efforts to track viral spread and assess zoonotic risks. Enhanced monitoring of predator-prey interactions and mammalian populations near avian habitats is recommended to mitigate future spillover events. Further in-depth genomic and phenotypic analyses are warranted to better understand the pathobiological characteristics and zoonotic potential of this virus.

## Data Availability

The datasets presented in this study can be found in online repositories. The names of the repository/repositories and accession number(s) can be found in the article/Supplementary material.

## Supplementary material

Supplementary Figure 1 Maximum-likelihood tree constructed using the complete coding nucleotide sequences of **(A)** polymerase basic protein 2, **(B)** polymerase basic protein 1, **(C)** polymerase acidic protein, **(D)** hemagglutinin protein, **(E)** nucleoprotein, **(F)** neuraminidase protein, **(G)** matrix protein, and **(H)** non-structural protein. Red taxa marked with circles represent the H5N1 isolate from the leopard cat (*Prionailurus bengalensis*) in South Korea. Purple taxa indicate the H5N1 HPAI viruses isolated from wild birds during the 2024–2025 winter season. Squares denote HPAI viruses detected in wild birds in South Korea in October 2024. Background shading indicates HPAI isolates in blue. Numerical values at the nodes represent bootstrap support values (%) based on 1,000 replicates. Bootstrap values below 70% are not shown.
